# Atlantoaxial Subluxation as the Initial Presentation of Rheumatoid Arthritis: A Case Report

**DOI:** 10.7759/cureus.52579

**Published:** 2024-01-19

**Authors:** Ryuichi Ohta, Junji Iwasa, Chiaki Sano

**Affiliations:** 1 Community Care, Unnan City Hospital, Unnan, JPN; 2 Orthopedic Surgery, Unnan City Hospital, Unnan, JPN; 3 Community Medicine Management, Shimane University Faculty of Medicine, Izumo, JPN

**Keywords:** rural health services, elderly, diagnostic imaging, primary health care, atlanto-axial subluxation, rheumatoid arthritis

## Abstract

Rheumatoid arthritis (RA) is known for its diverse manifestations, although atlantoaxial subluxation is a rare complication. This case report sheds light on the complexity of RA diagnosis, especially in the elderly, and emphasizes the significance of primary care in identifying atypical presentations. A 68-year-old male with a history of chronic obstructive pulmonary disease, hypertension, prior traumatic neck spinal injury, and lumbosacral stenosis presented to a rural community hospital with neck pain, bilateral numbness, and arm weakness. Initially diagnosed with degenerative cervical spondylosis, his condition later progressed to include swollen, painful wrists and metacarpophalangeal joints. Diagnostic tests revealed elevated rheumatoid factor and C-reactive protein, and hand X-ray showed bone erosions. An MRI confirmed an atlantoaxial subluxation. He was diagnosed with RA based on the American College of Rheumatology/European League Against Rheumatism 2010 criteria with a score of 7. The patient underwent cervical fusion surgery and rehabilitation, leading to significant functional improvement. This case underscores the importance of a comprehensive diagnostic approach in primary care for elderly patients presenting with non-specific symptoms. It highlights the need for heightened awareness among general practitioners of atypical RA manifestations, such as atlantoaxial subluxation. The case advocates for continued research into early detection and management strategies for such rare presentations to enhance patient outcomes in RA.

## Introduction

Rheumatoid arthritis (RA), with its prevalence ranging between 0.5% and 1%, is one of the more common and complex rheumatic diseases in clinical practice [1]. Characterized by chronic and systemic inflammation, RA extends its effects beyond the typical joint and musculoskeletal involvement, frequently affecting vital organs such as the heart, lungs, and neurological system [2]. The multifaceted impacts of RA significantly impair patients' physical well-being and quality of life, underscoring the need for prompt, accurate diagnosis and effective treatment strategies [3].

The clinical progression of RA is notably heterogeneous, displaying variability in its progression and the extent of joint involvement. While primarily affecting peripheral joints like the wrists, metacarpophalangeal joints, and proximal interphalangeal joints, RA can also manifest in more rare forms, involving axial skeletal structures such as the atlantoaxial joints, with a prevalence of only 17% in advanced RA cases [4]. These atypical presentations often present diagnostic challenges, necessitating a high level of suspicion and comprehensive disease understanding [5,6].

We report a case of an older patient initially presenting with numbness in both hands, a common symptom in general medical practice, which led to a diagnosis of RA complicated by atlantoaxial subluxation. This case highlights the diversity of RA's initial presentations and the complexities of diagnosing such atypical manifestations.

## Case presentation

Patient background

A 68-year-old male presented at a rural community hospital with chief complaints of persistent neck pain, bilateral numbness, and arm weakness lasting two months. Initially experiencing dull, continuous neck pain with gradual onset, the pain was worse in the mornings but improved with movement. One month before the presentation, he was diagnosed with degenerative cervical spondylosis at an orthopedic clinic and treated with acetaminophen (1,500 mg/day). Concurrently, he reported progressive numbness and weakness in both arms, particularly in the mornings. A week before hospital visitation, he experienced significant hand mobility impairment, affecting his independence. His medical history included chronic obstructive pulmonary disease (12 years), hypertension (11 years), a prior traumatic neck spinal injury (10 years ago), and lumbosacral stenosis (six years). Ongoing medications included valsartan (80 mg), amlodipine (5 mg), and tiotropium bromide hydrate.

Initial assessment

Vital signs at presentation were stable: a blood pressure of 135/84 mmHg, a pulse rate of 84 beats/min, a body temperature of 36.4°C, a respiratory rate of 18 breaths/min, and an oxygen saturation of 96% on room air. The patient was oriented and alert. Physical examination revealed bilateral muscular atrophy in the thenar, hypothenar, and lumbrical muscles, with tendon hyperreflexia in both arms. No other joint or neurological abnormalities, such as hyperreflexia and Babinski's reflex, were observed. Chest, abdominal, and skin examinations were unremarkable. Laboratory tests showed no elevation in inflammatory markers (Table 1).

**Table 1:** Initial laboratory data of the patient CK, creatine kinase; eGFR, estimated glomerular filtration rate

Parameter	Level	Reference
White blood cells	6.40	3.5–9.1 × 10^3^/μL
Neutrophils	62.2	44.0–72.0%
Lymphocytes	21.3	18.0–59.0%
Monocytes	12.8	0.0–12.0%
Eosinophils	3.3	0.0–10.0%
Basophils	0.4	0.0–3.0%
Red blood cells	5.21	3.76–5.50 × 10^6^/μL
Hemoglobin	15.9	11.3–15.2 g/dL
Hematocrit	47.4	33.4–44.9%
Mean corpuscular volume	90.9	79.0–100.0 fl
Platelets	24.3	13.0–36.9 × 10^4^/μL
Total protein	7.7	6.5–8.3 g/dL
Albumin	4.6	3.8–5.3 g/dL
Total bilirubin	7.7	0.2–1.2 mg/dL
Aspartate aminotransferase	23	8–38 IU/L
Alanine aminotransferase	14	4–43 IU/L
Alkaline phosphatase	78	106–322 U/L
γ-Glutamyl transpeptidase	101	<48 IU/L
Lactate dehydrogenase	202	121–245 U/L
Blood urea nitrogen	18.8	8–20 mg/dL
Creatinine	1.01	0.40–1.10 mg/dL
eGFR	56.9	>60.0 mL/min/L
Serum Na	138	135–150 mEq/L
Serum K	4.4	3.5–5.3 mEq/L
Serum Cl	106	98–110 mEq/L
Serum Ca	10.2	8.8–10.2 mg/dL
Serum P	2.8	2.7–4.6 mg/dL
Serum Mg	2.2	1.8–2.3 mg/dL
CK	90	56–244 U/L
CRP	0.23	<0.30 mg/dL

Neck X-ray revealed no fractures or spinal cord compression. The patient was prescribed pregabalin (150 mg/day), and his symptoms were alleviated but still existed.

Subsequent developments

**Figure 1:**
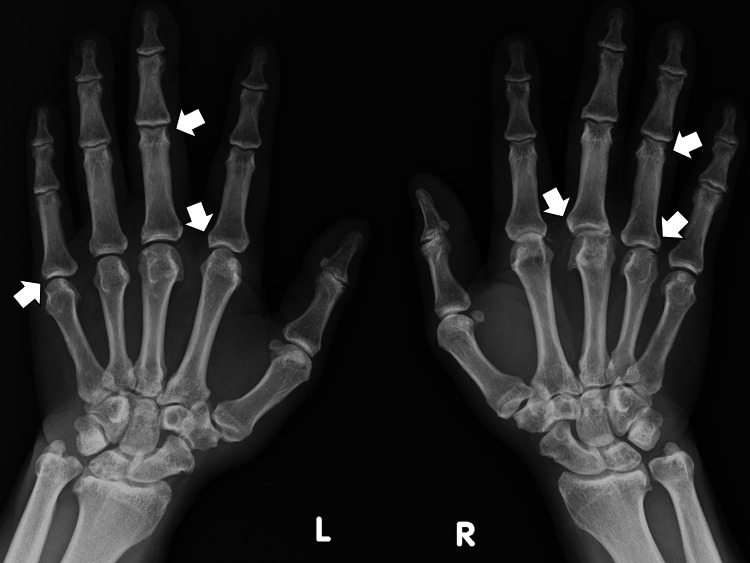
A hand X-ray showing several bone erosions in the affected joints (white arrows)

One month later, the patient developed continuous swelling and pain in the bilateral wrists and metacarpophalangeal joints, accompanied by morning stiffness. A hand X-ray showed bone erosions in the affected joints (Figure 1).

**Figure 2:**
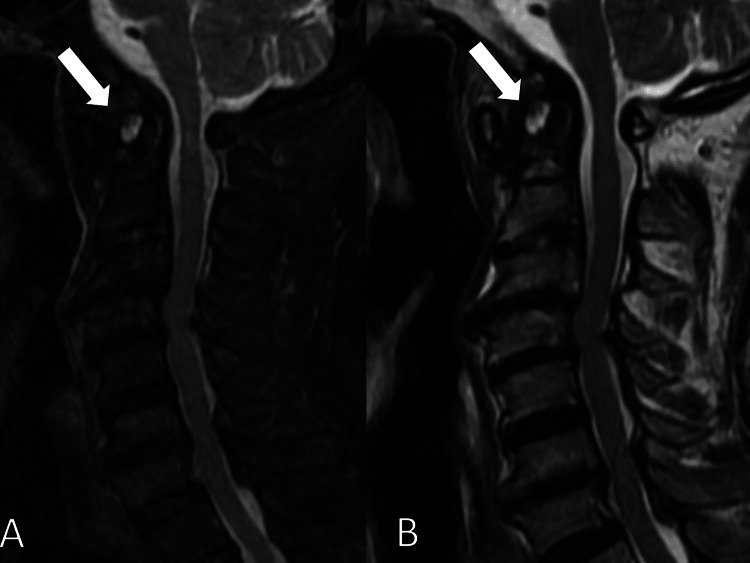
MRI of high signal areas of dens, showing the inflammation of the dens axis (white arrows) (A) T2 enhanced; (B) short-tau inversion recovery

Additional laboratory tests revealed high titers of rheumatoid factor (72 IU/mL; reference <15) without any positivity of antinuclear antibodies or anti-citrullinated protein antibodies, coupled with high C-reactive protein (0.82 mg/dL; reference <0.3). He met the American College of Rheumatology/European League Against Rheumatism 2010 criteria for RA (score of 7). Deteriorating neck and arm pain led to an MRI, which showed inflammation of the dens axis and a positive instability test of the atlantoaxial joint, indicative of atlantoaxial subluxation secondary to RA (Figure 2).

Management and outcome

The patient was referred to a university hospital for surgical intervention following interdisciplinary discussions. He underwent cervical fusion surgery, followed by rehabilitation, resulting in significant improvement and eventual discharge with restored daily activity functionality.

## Discussion

This case report delineates a rare but significant presentation of atlantoaxial subluxation in an elderly patient with RA. RA typically presents with symmetrical polyarthritis, predominantly affecting the small joints of the hands and feet. However, as this case illustrates, the involvement of the atlantoaxial joint, while less common, underscores the systemic and unpredictable nature of RA. According to the case, there can also be RA with an undiagnosed presentation with months of evolution without treatment until reaching the presentation of atlantoaxial subluxation. The literature indicates that while atlantoaxial subluxation occurs in a minority of RA patients, it can lead to severe complications, including neurological deficits and sudden death due to spinal cord compression [4].

The expansive nature of RA's joint involvement emphasizes the need for a comprehensive diagnostic approach. In this case, the patient had multiple chronic diseases, often managed by general physicians as well as system-specific specialists. This scenario is common in rural settings, where general physicians frequently encounter undifferentiated symptoms that may relate to rheumatic diseases [7-9]. Therefore, rural general physicians should be aware of RA's potential to affect various joints, including less traditionally associated joints like the atlantoaxial joints [10]. This case highlights the importance of considering RA in the differential diagnosis when patients present with non-specific arthritic symptoms.

Atlantoaxial subluxation in RA patients can be a critical condition, potentially impacting the quality and longevity of life. If not identified and managed early, atlantoaxial subluxation can lead to permanent spinal damage as the disease progresses [11]. Early recognition and appropriate management are crucial to prevent severe outcomes such as paraplegia and severe neurological pains [12]. In our case, the development of arthritis in the patient enabled the diagnosis of atlantoaxial subluxation. General physicians must be vigilant in their examination of joints to detect such rare presentations of RA [13].

The management of RA patients with atlantoaxial subluxation necessitates a multidisciplinary approach. Collaboration between general physicians, rheumatologists, and orthopedic surgeons is essential for achieving optimal patient outcomes, especially in rural community hospitals [14]. In rural settings where rheumatologists may not be readily available, constant communication between general physicians and orthopedic surgeons is crucial for diagnosing conditions like atlantoaxial subluxation [15,16]. Treatment strategies can vary from conservative management, such as cervical immobilization and antirheumatic drugs, to surgical interventions in more advanced cases [17,18]. General physicians in rural contexts should be familiar with managing RA and its medications to prevent its progression [19]. Orthopedic surgeons need to collaborate with general physicians and plan surgeries to prevent the progression of symptoms [20]. Decisions should be individualized, considering the patient's overall health, subluxation severity, and neurological symptoms.

## Conclusions

This case report highlights the critical role of general physicians in recognizing and managing the diverse presentations of RA, particularly in elderly patients. It demonstrates the necessity for primary care providers to maintain a high level of clinical suspicion for atypical RA manifestations, such as atlantoaxial subluxation. This case underscores the importance of ongoing education and training for generalists in the early detection and referral strategies for complex RA cases.
